# Refugee camp health services utilisation by non-camp residents as an indicator of unaddressed health needs of surrounding populations: a perspective from Mae La refugee camp in Thailand during 2006 and 2007

**DOI:** 10.11604/pamj.2019.32.188.16780

**Published:** 2019-04-17

**Authors:** Lykourgos Christos Alexakis, Maria Athanasiou, Angeliki Konstantinou

**Affiliations:** 1 Médecins Sans Frontières, 15 Xenias Street, 11527 Athens, Greece; 2 Première Urgence-Aide Médicale Internationale, Mae Sot, Tak 63110, Thailand; 3 National School of Public Health, 196 Alexandras Avenue, 11521 Athens, Greece; 4 Hospital zum Heiligen Geist, Lange Str. 4-6, 60311 Frankfurt am Main, Germany

**Keywords:** Refugee camps, health services, host community, host population

## Abstract

**Introduction:**

This study explored the differences on the level of medical care required by camp and non-camp resident patients during utilisation of the health services in Mae La refugee camp, Tak province, Thailand during the years 2006 and 2007.

**Methods:**

Data were extracted from camp registers and the Health Information System used during the years 2006 and 2007 and statistical analysis was performed.

**Results:**

The analysis showed that during 2006 and 2007 non-camp resident patients, coming from Thailand as well as Myanmar, who sought care in the outpatient department (OPD) of the camp required at a significantly higher proportion admission to the inpatient department (IPD) or referral to the district hospital compared to camp resident patients. Although there was a statistically significant increased mortality of the non-camp resident patients admitted in the IPD compared to camp resident patients, there was no significant difference in mortality among these two groups when the referrals to the district hospital were analysed.

**Conclusion:**

Non-camp resident patients tended to need a more advanced level of medical care compared to camp resident patients. Provided that this it is further validated, the above observed pattern might be potentially useful as an indirect indicator of unaddressed health needs of populations surrounding a refugee camp.

## Introduction

Mae La Refugee camp (Tak Province, Thailand) was originally established in 1984. As of end of June 2007 the camp had a population of 49,783 [1]. Since 2005 the non-governmental organisation (NGO) *Aide Médicale Internationale* (AMI) was responsible for all aspects of medical care in the camp with the exception of the tuberculosis program which was taken care by *Médecins Sans Frontières* (MSF) and obstetrics care which was managed by Shoklo Malaria Research Unit (SMRU). The health facilities run by AMI consisted of two Out-Patient Departments (OPD), one 154-bed In-Patient department (IPD) as well as two laboratories and a pharmacy stock, all located within the refugee camp. All these departments were staffed by refugees trained to work independently as medics (performing medical acts), nurses or lab technicians. In addition to them there were minimum one to maximum two full time expatriate medical doctors specifically assigned to clinical supervision responsibilities who were providing daily support to the refugee staff.

Most of the patients were dealt with in the OPD, while the complicated cases were hospitalised in the IPD. Selected patients were referred to Mae Sot General Hospital (MSGH) which was the district hospital and was located on a distance approximately one hour drive by car from the camp. These patient referrals were mainly for surgical operations, radiology exams or specialist consultations. Samples for laboratory tests which could not be performed in the camp's lab were also sent there. Mae La facilities constituted an example of a basic health care system which involved versatile health workers able to cope with a variety of health problems [2]. Delivery of health care and drugs was free of charge for all patients independent of their status being camp residents or not. One of the authors (LCA) while working in the camp during 2007-2008 observed that patients who were not camp residents, presented in a worse general health status compared to camp residents. They suffered from more complicated morbidity often neglected for long time and appeared to represent a considerable and disproportionate part of the staff's workload. These patients were coming from Myanmar side of the border, as well as from Thai villages in the vicinity. By contrast camp residents (defined as people living in the camp for more than three months continuously and independently of whether they were UNHCR registered refugees or not) appeared to be in better general health status. In order to quantify the burden of non-camp resident patients on the camp's health care facilities a detailed review of the available anonymous data was organized. The research question was whether there was a difference between camp and non-camp residents on the level of medical care, they required during utilisation of the health services available in Mae La refugee camp.

## Methods

This study was a purely descriptive observational evaluation based on routinely collected anonymous and non-identifiable population data. Figures on OPD consultations and IPD admissions for camp residents as well as non-camp residents were extracted from the Health Information System (HIS) used by Aide Médicale Internationale in Mae La refugee camp for the years 2006 and 2007. Figures concerning referrals to Mae Sot General Hospital for the same years (2006 and 2007) of camp and non-camp resident patients were obtained from the camp's referrals register. Mortality figures in the IPD as well as among referrals to the district hospital for camp and non-camp resident patients were extracted from the HIS for the years 2006 and 2007. Statistical analysis was performed by the use of STATA software (version 10.1). The absolute numbers and percentages of non-camp residents in the total number of OPD consultations, IPD admissions, and referrals to the district hospital were calculated per month and subsequently were used to calculate the values for 2006 and 2007 respectively. The association between the origin of the patients (camp or non-camp) and the different levels of care that was required (OPD consultation, IPD admission, referral to district hospital) was tested using chi-square test. P-value < 0.001 was considered statistically significant. For IPD admissions and for hospital referrals, the association between the origin of the patients and the final outcome (death or not death) was tested by means of Fisher's exact test.

## Results

During the year 2006 non-camp residents comprised 3.75% of all OPD consultations, 8.2% of all IPD admissions and 15.91% of all referrals to the Thai hospital. The same pattern was also evident for 2007 with non-camp residents comprising 3.16% of OPD, 7.78% of IPD and 12.52% of referrals (Table 1). Specifically for the year 2007 we had also detailed data on the origin of non-camp resident patients. From the total OPD consultations of non-camp resident patients during this year (n=3814), 37.86% (n=1444) came from Myanmar (Burma), 60.59% (n= 2311) came from Thailand and 1.54% (n=59) concerned follow up consultations in which the exact origin of the non-camp resident patient was not registered again. From all the non-camp resident patients who were admitted in Mae La IPD during 2007 (n=539), 51.39% (n=277) came from Myanmar while 48.61% (n=262) came from Thailand. From the non-camp resident patients referred to the district hospital during the same year (n=61), 80.33% (n=49) came from Myanmar while 19.67% (n=12) came from Thailand (Table 2). During the year 2006 IPD mortality for non-camp resident patients was 34.63/1000 admissions (25 deaths out of 722 admissions) and for camp resident patients was 8.67/1000 admissions (70 of 8078). The same year the mortality among referrals to the district hospital for non-camp resident patients was 21.74/1000 admissions (2 of 92) and for camp resident patients was 41.15/1000 admissions (20 of 486). During 2007 IPD mortality for non-camp resident patients was 57.51/1000 admissions (31 deaths out of 539 admissions) and for camp resident patients was 11.58/1000 admissions (74 of 6390). The same year the mortality among referrals to the district hospital for non-camp resident patients was 81.97/1000 admissions (5 of 61) and for camp resident patients was 61.03/1000 admissions (26 of 426) (Table 3). The association between the origin of the patient (camp or non-camp resident) and the different levels of care that was needed was tested using chi-square test. When using the 2006 data there was a statistically significant difference between the origin of patients and the level of care they needed (Pearson chi2=619.38, P=0.000). Non-camp resident patients tended to need a more advanced level of medical care compared to camp resident patients. Similar results were obtained when data regarding 2007 were used (Pearson chi2=539.39, P=0.000). During 2007 too there was a statistically significant difference between the origin of patients and the level of care needed and non-camp resident patients tended to need a more advanced level of medical care compared to camp resident patients.

**Table 1:** Proportion of non-camp residents in the total number of medical acts through different levels of medical care in Mae La refugee camp during 2006 and 2007

Non-Camp Residents	2006	2007
**OPD** consultations	**3.75%** n=4,620 out of a total of 123,067 95%CI: 3.65%-3.86%	**3.16%** n=3,814 * out of a total of 120,490 95%CI: 3.07%-3.27% (* = consisting of 3,755 new and 59 follow up consultations. Similar data were not available for the year 2006)
**IPD** admissions	**8.2%** n=722 out of a total of 8,800 95%CI: 7.64%-8.80%	**7.78%** n=539 out of a total of 6,929 95%CI: 7.16%-8.43%
**Referrals** to district hospital	**15.91%** n=92 out of a total of 578 95%CI: 13.03%- 19.16%	**12.52%** n=61 out of a total of 487 95%CI: 9.72%- 15.80%

**Table 2:** Non-camp resident patients during 2007 per country of origin

	Myanmar (Burma)	Thailand
OPD new consultations	1,444	2,311
IPD admissions	277	262
Referrals to hospital	49	12

**Table 3:** Fatal cases per 1000 admissions for IPD and referrals

	2006	2007
IPD non-camp	34.63	57.51
IPD camp	8.67	11.58
Referrals non-camp	21.74	81.97
Referrals camp	41.15	61.03

IPD: inpatient department

In addition, for 2007 the association between the countries of origin (Myanmar or Thailand) within the non-camp resident patients group was also tested as data were available for this year (Table 2). A statistically significant difference between the two subgroups was found, with non-camp resident patients coming from Myanmar tending to need more advanced level of medical care compared to non-camp resident patients coming from Thailand (Pearson chi2=73.083, P=0.000). The association between the origin of patients and the final outcome (death or not death) was tested using Fisher's exact test. Regarding IPD admissions in 2006, there was a statistically significant increased mortality in non-camp resident patients compared to camp resident patients (Fisher's exact P=0.000). The final outcome of cases that were referred to the district hospital in 2006 was not associated with their origin, that is there was no statistically significant difference in mortality between non-camp and camp resident patients who were referred to the district hospital (Fisher's exact P=0.5546). Similar results were obtained when we used data regarding 2007. For IPD admissions in 2007 there was a statistically significant increased mortality in non-camp resident patients compared to camp resident patients (Fisher's exact P=0.000). Also during this year there was no statistically significant difference in mortality between non-camp and camp resident patients referred to the district hospital (Fisher's exact P=0.5718).

## Discussion

**Figure 1:**
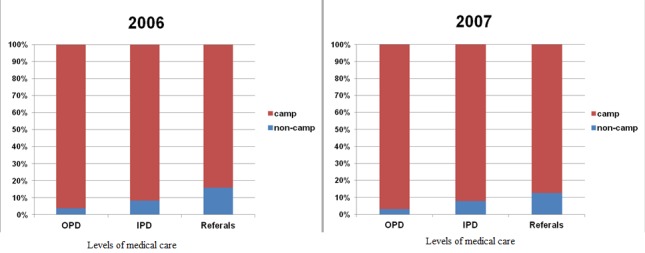
Percentage of camp resident patients and non-camp resident patients in the different levels of medical care during the years 2006 and 2007 in Mae La refugee camp, Tak province, Thailand. OPD: outpatient department; IPD: inpatient department

As shown in Table 1 there was a statistically significant increase in the portion of non-camp resident patients in each level of care as we proceed from OPD consultations to IPD admissions and to district hospital referrals during 2006 and 2007 in Mae La refugee camp. Non-camp resident patients tended to need a more advanced level of medical care compared to camp resident patients during the same period. This pattern (Figure 1) might reflect a worse general health status as well as more complicated or neglected for long time morbidity of non-camp residents compared to camp residents. It could potentially indicate unaddressed health needs of surrounding populations within Myanmar as well as within Thailand during the period studied. Globally it is common for refugee camp health facilities to provide care also to non-camp residents. On average 2% of OPD consultations in Asia and 21% in Africa were attributable to host community members [3]. Many different reasons might have influenced health care seeking behaviour of non-camp residents in the case of Mae La camp. Proximity to the camp could have been a contributing factor as certain Thai villages (eg. Ban Mae Ok Hu, Ban Mae La) were located closer to the camp's medical facilities than the closest public Thai hospital. The camp was located just next to a major paved road connecting the border towns of Mae Ramat and Tha Song Yang, so it was easily accessible. Language was another factor as many non-camp resident patients were ethnic Karen and they spoke Karen and Burmese. Communication difficulties for non-Thai speakers while seeking care in Thai health system could lead to frustration or dissatisfaction. By contrast most of the staff who worked in the camp medical facilities spoke Karen, Burmese, English and often also Thai. During 2007 in the two districts around Mae La refugee camp (Tha Song Yang and Mae Ramat districts) the migrant Burmese population with all ethnicities included was estimated to be 30,405 out of a total population of 137,020 (excluding the refugee camp population). Many of these migrants were not officially registered and had no access to social security. During 2007 only 17,633 migrants had social security in the whole Tak province [4]. Uninsured migrants who were living in Thailand as well as patients who were arriving from the Myanmar side of the border might have been more motivated to use the health care services available in Mae La camp, as medical care there as well as any potential referral to a Thai hospital was provided free of charge. Internationally displaced people remain a disadvantaged population even if they become integrated in the local host community. This has been demonstrated in rural South Africa where higher childhood mortality rates were found among children from former Mozambican refugee households compared to those from the host community [5]. In a study among refugees living in Pretoria, South Africa several challenges have been identified. These included the luck of security, language barriers, the difficulty of obtaining legal papers, the luck of employment, deplorable living conditions and falling victims of crime or police harassment [6]. On the other hand in Guinea, refugees were allowed to settle in existing villages instead of refugee camps and were given free access to national health services, which were reinforced. The improvement of the health system and transport infrastructure was reflected in an increase of the rates of major obstetric interventions for the host population [7].

When trying to explain the increased morbidity of non-camp resident patients compared to camp resident patients we have to acknowledge the situation in Myanmar at the same time period. At that time there were still ongoing conflicts in the eastern provinces of the country having a direct effect on the health status of the affected communities. For populations living in conflict zones in eastern Burma, high crude, under-five and infant mortality rates have been described [8]. In Karen state as of October 2007 the number of Internally Displaced Persons was estimated at 116,900 [9]. Conflict and displacement might further decrease access of the affected population to primary and secondary care as well as essential drugs, in a country where the per capita government expenditure on health is already very low. In Myanmar this was at 4.9 international dollar rate in 2004 compared to Thailand at 189.7 for the same year [10]. Different approaches have been used to improve the health outcomes in this region, one of which included the training of village health workers to implement malaria control interventions among the internal displaced populations [11]. In addition to local backpack health worker teams supported by cross border local-global partnerships, mobile obstetric maternal health workers have been providing some health services to internally displaced persons in eastern Burma [12, 13]. Nevertheless a considerable number of patients was crossing the border between Myanmar and Thailand in order to obtain health care when this was unavailable or unaffordable locally [14, 15]. On the other hand Mae La refugee camp was a stable protected environment located within Thailand, where many aspects of human security were catered for [16]. Situated in short distance, eight kilometers, from the Thai-Myanmar border it was accessible on foot. The camp provided decent medical facilities with health care workers who could speak both Karen and Burmese. Medical care, basic lab exams and quality drugs were provided free of charge. A patient arriving there would receive free primary outpatient or inpatient care according to his needs and had many chances to be reviewed by a qualified medical doctor, or even referred for specialised exams or a surgical operation in a well equipped Thai hospital, with all expenses paid by NGOs. A refugee camp may develop *de facto* and out of necessity into an important health care provider and a reference health care facility for unprivileged populations in its vicinity besides the refugee population itself. This is easier during a stable, chronic, post-emergency phase especially when the inpatient and outpatient departments within the camp are well supported and equipped by different actors, and the possibility of district hospital referral exists, as it was the case in Mae La. Its function can be complementary to existing state health facilities as it may provide care to populations with limited access to the health system of the host country as well as to individuals arriving from conflict affected areas of a neighboring country. Although the ideal model of health service delivery for refugees and host population is not yet clear, analysis of utilisation patterns for refugees and host population might help future policy planning in this field [17].

Several limitations exist in this study. It is possible that only poor non-camp patients from the host community sought care in the camp. Compromised health status due to malnutrition, bad hygiene or poor living conditions as consequences of poverty could reflect in our data. Medics might have been more willing to admit in the IPD a non-camp resident patient who was unable to stay overnight in the camp in situations where outpatient follow up would have been preferred for camp residents. Selection bias during the referral procedure could also occur. There were budget constrains as transport, examinations and hospitalisation costs for all patients referred to the district hospital were paid by AMI. As a result the majority of referrals, from both patient groups, were acute surgical referrals (e.g. fractures, trauma patients, appendicitis, intestinal obstruction, pyomyositis) which could be dealt with quickly and cost effectively with a good prognosis. Acute and chronic medical problems, infections, malaria, HIV/AIDS, blood transfusions, cancer and terminally ill patients were taken care in the OPD and IPD of the camp with the means available there. This could influence and partly explain the difference observed in mortality among the two groups in IPD admissions and its absence among those referred to hospital. Another aspect is the exact geographic distribution by origin of the non-camp resident patients who sought care in Mae La camp. Although not covered in this study, it might have been useful to know precisely from which villages within Thailand or Myanmar these patients came. Such a mapping could point to specific geographic areas in both sides of the border with poor access to primary and/ or secondary health care. Due to lack of data regarding the number and geographic distribution of host and migrant populations in Thailand as well as of populations in the Myanmar side of the border, the frequency that non-camp resident populations used the health services of Mae La camp could not be calculated in this study. Although the data in this study are relatively old, they can provide a historical perspective of the situation during the chronic stable phase of one of the biggest refugee camps in the Thailand-Myanmar border area during the years 2006 and 2007. This information can be useful for comparisons with different geographic areas or newer data in other studies. Furthermore from 2008 onwards a new HIS for data collection was implemented in the camp. This was introduced by United Nations High Commissioner for Refugees (UNHCR) in order to standardise data collection across refugee camps. The new HIS differentiated only two groups of patients: refugee versus host country patients. As a result information concerning the detailed origin of non-camp resident patients, which is documented in this study, was not routinely collected anymore [18].

## Conclusion

A considerable number of OPD consultations in Mae La refugee camp during 2006 and 2007 involved patients who were non-camp residents, originating from surrounding Thai villages and the Myanmar side of the border. These patients required at a statistically significant higher proportion admission to the IPD of the camp or referral to the district hospital in comparison with camp residents. Non-camp resident patients admitted in IPD suffered from a statistically significant increased mortality compared to camp residents admitted in the same department. This pattern of refugee camp health services utilisation by non-camp residents might reflect a worse health status of the non-camp resident population compared to the camp resident one, possibly indicating unaddressed health needs in this population. The health facilities of a refugee camp during the chronic stable phase might function as a reference health facility for populations from the surrounding area who might have difficulties in accessing other health care facilities. Further research in this field is needed in order to map any unaddressed health needs in the Thailand-Myanmar border area and provide an update of the current situation. This could be subsequently compared with the pattern observed during 2006 and 2007 described in this study. Provided that this pattern is further validated in similar settings in other geographic areas, it might prove a useful indirect indicator of unaddressed health needs of surrounding populations living in proximity to refugee camps or similar settlements.

### What is known about this topic

- From the OPD consultations taking place in refugee camps, 2% in Asia and 21% in Africa on average are attributable to host community members;
- In some cases refugee hosting improved the quality and accessibility of health services and health outcomes for host national population but there are limited data to support integrated health services;
- Analysis of health services utilization patterns for refugees and host population might help future policy planning.

### What this study adds

- A refugee camp health services utilisation pattern in which non-camp resident patients visiting the OPD require at a significantly higher proportion admission to the IPD or referral to the district hospital, compared to camp resident patients, might indicate unaddressed health needs of populations living in the area around the refugee camp;
- A pattern where non-camp resident patients admitted to the IPD present with significantly increased mortality compared to camp resident patients might also indicate unaddressed health needs in the surrounding area;
- These health services utilisation patterns observed in a major refugee camp in the Thailand-Myanmar border area during 2006-2007 should be further explored in similar settings in other geographic areas.

## Competing interests

The authors declare no competing interests.

## References

[1] Aide Médicale Internationale (2007). Mae La Camp Health Information System, Demography.

[2] Criel B, Kegels G, Van der Stuyft P (2004). Editorial: a framework for analyzing the relationship between disease control programmes and basic health care. Tropical Medicine and International Health.

[3] Weiss W, Vu A, Tappis H, Meyer S, Haskew C, Spiegel P (2011). Utilization of outpatient services in refugee settlement health facilities: a comparison by age, gender, and refugee versus host national status. Conflict and Health.

[4] Mae Sot General Hospital (2008). Personal communication from Department of Social Medicine. Mae Sot General Hospital.

[5] Hargreaves J, Collinson M, Kahn K, Clark S, Tollman S (2004). Childhood mortality among former Mozambican refugees and their hosts in rural South Africa. International Journal of Epidemiology.

[6] Rugunanan P, Smit R (2011). Seeking refuge in South Africa: challenges facing a group of Congolese and Burundian refugees. Development Southern Africa.

[7] Van Damme W, De Brouwere V, Boelaert M, Van Lerberghe W (1998). Effects of a refugee-assistance programme on host population in Guinea as measured by obstetric interventions. The Lancet.

[8] Lee TJ, Mullany LC, Richards AK, Kuiper HK, Maung C, Beyrer C (2006). Mortality rates in conflict zones in Karen, Karenni, and Mon states in eastern Burma. Tropical Medicine and International Health.

[9] Thailand Burma Border Consortium (TBBC) (2007). Internal Displacement in Eastern Burma-2007 Survey.

[10] World Health Organisation(WHO) Countries.

[11] Lee CI, Smith LS, Shwe Oo EK, Scharschmidt BC, Whichard E, Kler T (2009). Internally displaced human resources for health: villager health worker partnerships to scale up a malaria control programme in active conflict areas of eastern Burma. Glob Public Health.

[12] Mahn M, Maung C, Oo EK, Smith L, Lee CI, Whichard E (2008). Multi-level partnerships to promote health services among internally displaced in eastern Burma. Glob Public Health.

[13] Mullany LC, Lee CI, Paw P, Shwe Oo EK, Maung C, Kuiper H (2008). The MOM Project: delivering maternal health services among internally displaced populations in eastern Burma. Reprod Health Matters.

[14] Hemhongsa P, Tasaneeyapan T, Swaddiwudhipong W, Danyuttapolchai J, Pisuttakoon K, Rienthong S (2008). TB, HIV-associated TB and multidrug-resistant TB on Thailand's border with Myanmar, 2006-2007. Trop Med Int Health.

[15] Tschirhart N, Sein T, Nosten F, Foster AM (2016). Migrant and refugee patient perspectives on travel and tuberculosis along the Thailand-Myanmar border: a qualitative study. PLoS One.

[16] Gutlove P, Thompson G (2003). Human security: Expanding the scope of public health. Medicine, Conflict and Survival.

[17] Meyer S, Tappis H, Weiss W, Spiegel P, Vu A (2011). Refugee site health service utilization: more needs to be done. Am J Disaster Med.

[18] UNHCR Health Information System, iRHIS, Integrated Refugee Health Information System Refugee health data.

